# Longitudinal Links between Executive Function, Anger, and Aggression in Middle Childhood

**DOI:** 10.3389/fnbeh.2018.00027

**Published:** 2018-02-27

**Authors:** Helena L. Rohlf, Anna K. Holl, Fabian Kirsch, Barbara Krahé, Birgit Elsner

**Affiliations:** Department of Psychology, University of Potsdam, Potsdam, Germany

**Keywords:** executive function, anger, relational aggression, physical aggression, reactive aggression, proactive aggression, childhood, longitudinal study

## Abstract

Previous research has indicated that executive function (EF) is negatively associated with aggressive behavior in childhood. However, there is a lack of longitudinal studies that have examined the effect of deficits in EF on aggression over time and taken into account different forms and functions of aggression at the same time. Furthermore, only few studies have analyzed the role of underlying variables that may explain the association between EF and aggression. The present study examined the prospective paths between EF and different forms (physical and relational) and functions (reactive and proactive) of aggression. The habitual experience of anger was examined as a potential underlying mechanism of the link between EF and aggression, because the tendency to get angry easily has been found to be both a consequence of deficits in EF and a predictor of aggression. The study included 1,652 children (between 6 and 11 years old at the first time point), who were followed over three time points (T1, T2, and T3) covering 3 years. At T1, a latent factor of EF comprised measures of planning, rated via teacher reports, as well as inhibition, set shifting, and working-memory updating, assessed experimentally. Habitual anger experience was assessed via parent reports at T1 and T2. The forms and functions of aggression were measured via teacher reports at all three time points. Structural equation modeling revealed that EF at T1 predicted physical, relational, and reactive aggression at T3, but was unrelated to proactive aggression at T3. Furthermore, EF at T1 was indirectly linked to physical aggression at T3, mediated through habitual anger experience at T2. The results indicate that deficits in EF influence the later occurrence of aggression in middle childhood, and the tendency to get angry easily mediates this relation.

## Introduction

A meta-analysis that included a wide range of EF measures concluded that EF is negatively associated to antisocial behavior, with varying effect sizes depending on the specific form of antisocial behavior and the occurrence of comorbid problems (Ogilvie et al., 2011). However, there is a lack of longitudinal studies that examined the effect of deficits in EF on the development of aggression, particularly in middle childhood, taking into account different forms and functions of aggression. The present study extends previous research by examining the longitudinal links between EF and different forms (relational and physical) and functions (reactive and proactive) of aggression over 3 years. In addition, previous research has mostly studied direct links between EF and aggression without considering potential underlying mechanisms. The present study addressed this issue by including individual differences in the experience of anger as a mediating variable.

### Executive function

There is disagreement in the literature over the exact definition of EF. However, EF can be described as an umbrella term that is usually equated with conscious, higher order processes associated with the prefrontal cortex (Hughes et al., 2005). EF governs goal-directed action and planning of behavior, and allows for adaptive responses to novel, complex, or ambiguous situations. As an important aspect of self-regulation, EF is considered vital for autonomous and adaptive psychological functioning (Séguin et al., 2007). Miyake et al. (2000) differentiated between three components of EF in college students, namely inhibition of prepotent responses, working memory updating, and mental set shifting. In a latent-variable analysis, these factors were moderately correlated, but clearly separable, and also had some common underlying mechanisms that contributed to all EF tasks. This unity-but-diversity framework is the most accepted conceptualization of EF, supported also by studies with children and adolescents (e.g., Lehto et al., 2003; Huizinga et al., 2006). *Inhibition* involves withholding or restraint of a motor response, and is considered central to EF (Miyake et al., 2000). Working memory updating (*working memory*) is the ability to maintain and manipulate information over brief periods of time (Huizinga et al., 2006). *Shifting* is the ability to alternate between mental rule sets or tasks (Miyake et al., 2000), and is considered the most complex EF component. An additional EF component that is also frequently mentioned is *planning*, which is essential to the EF domains of goal setting and goal-oriented behavior (Anderson, 2002). Unlike other cognitive abilities, EF shows a pronounced development after early childhood, paralleling the protracted maturation of the prefrontal cortex (Blakemore and Choudhury, 2006). The single components of EF, however, seem to follow differential courses throughout childhood and adolescence, involving progressive and regressive phases of development (Best et al., 2009; Best and Miller, 2010).

### Aggression

Among the many advantages of EF is the ability to regulate behavior that is prohibited by social norms, such as aggressive behavior. Aggression is defined as “any form of behavior directed toward the goal of harming or injuring another living being that is motivated to avoid such treatment” (Baron and Richardson, 1994, p. 7). In the present study, we distinguished between different forms and functions of aggression. A widely used classification of forms of aggressive behavior is the distinction between physical and relational aggression. *Physical aggression* refers to behavior that is intended to harm another person through the threat or use of physical force, whereas *relational aggression* is defined as behavior aimed at damaging another person's social relationships or feeling of social inclusion (Crick and Grotpeter, 1995). Children's use of physical aggression normally decreases during their preschool years, whereas relational aggression tends to increase during middle childhood, particularly in girls (Côté et al., 2007).

The distinction between different functions refers to the motivation that leads a person to act aggressively. Unprovoked aggressive behavior that aims to reach a certain goal, such as social dominance or the achievement of material goals, is described as *proactive aggression*. Proactively aggressive behavior can also be described as “offensive,” “instrumental,” and “cold-blooded” (Vitaro et al., 2006). Proactive aggression is conceptually linked to callous-unemotional (CU) traits. Children high on CU traits are characterized by a lack of guilt, reduced empathy, reduced display of emotions, callousness, and uncaring behavior (Vitaro et al., 2006; Blair et al., 2014), and they use aggressive behavior to reach desired rewards or social dominance (Pardini et al., 2003). *Reactive aggression*, by contrast, refers to aggressive behavior that is displayed in response to a perceived threat or provocation (Dodge and Coie, 1987; Card and Little, 2006). Reactively aggressive behavior can also be described as “defensive,” “impulsive,” and “hot-blooded” (Walters, 2005). The majority of children seem to follow a stable-low course in reactive and proactive aggression over the course of middle childhood, but some children show substantial changes by either increasing or decreasing their use of reactive and/or proactive aggression (Cui et al., 2016). Taken together, longitudinal evidence suggests that middle childhood is a period of important developmental change for both the forms and functions of aggression.

### The link between executive function and aggression

A large body of correlational research has shown that EF is negatively related to aggression in preschool-aged children and adolescents. For instance, low levels of EF coincide with preschoolers' externalizing behavior, which includes aggressive behavior (e.g., Hughes and Ensor, 2008). Similarly, preschoolers who were rated as “hard to manage” by their parents showed significantly lower EF than a less problematic comparison group (Hughes et al., 1998). A meta-analysis that covered a broad age range from early childhood to adulthood concluded the negative relation between EF and antisocial behavior to be robust, with one of the largest effects for externalizing behavior disorder (Ogilvie et al., 2011). Similarly, in a meta-analysis on preschoolers, EF, inhibition in particular, was correlated with externalizing behavior with a medium effect size (Schoemaker et al., 2013). Longitudinal evidence demonstrated that 3-year old children with low levels of effortful control, a cognitive construct closely related to EF (Bridgett et al., 2013), showed an increased risk for a chronic pattern of elevated externalizing behavior throughout middle childhood (Olson et al., 2017). By comparison, research on the relation between EF and aggression in middle childhood is limited, although this age range would be important to investigate. As a consequence of the developmental change in the forms (Côté et al., 2007) and functions (Cui et al., 2016) of aggression in middle childhood and the ongoing development of EF (e.g., Blakemore and Choudhury, 2006), this developmental period is of particular interest.

Different theoretical explanations have been proposed for the link between EF and aggression. The frontal-lobe hypothesis of emotional and behavioral regulation suggests that cognitive-neuropsychological functions in the frontal lobe, which are related to EF, appear to be systematically impaired in individuals showing physical aggression (Séguin, 2009). That is, children whose physically aggressive behavior does not decline after preschool as expected (Côté et al., 2007) are thought to have deficits in their EF. As a consequence, they have problems in regulating their behavior and solving social problems (Séguin and Zelazo, 2005; Zadeh et al., 2007). For example, they may not represent a problem adequately, may show deficits in planning a solution, or in reacting flexibly to different kinds of social situations. This is also the focus of another theoretical framework, the Social Information Processing (SIP) model, which proposes that children who show aggressive behavior may have deficits in their social information processing compared to nonaggressive age mates (Crick and Dodge, 1994). These difficulties may be influenced by children's EF (Huesmann et al., 1987; de Castro and van Dijk, 2018).

A further explanation relates to the integration of emotional processes into social-cognitive information processing (Lemerise and Arsenio, 2000). Particularly, anger seems to play an important part in the mediation between social-cognitive processes and aggressive responses (de Castro et al., 2005). EF is involved in the regulation of negative affect already in toddlers (Putnam et al., 2008) and preschool children (Carlson and Wang, 2007). Accordingly, deficits in EF may increase the experience of anger. Indeed, deficits in EF are linked to higher levels of negative affect, including anger (Gagne and Hill Goldsmith, 2011; Healey et al., 2011; Bridgett et al., 2013). In addition, higher cognitive control—a related construct to EF—in adolescence seems to act as a buffer against later, maladaptive outcomes of chronic anger, for example, adult antisocial personality traits (Hawes et al., 2016). Further evidence comes from research on irritability, which is defined as an increased proneness to anger (Leibenluft, 2017). That is, children with higher levels of irritability showed deficits in the processing of emotional stimuli, impaired context-sensitive regulation (Leibenluft and Stoddard, 2013), and neural dysfunction in processes associated to EF, such as error monitoring, reward processing, and emotion regulation (Perlman et al., 2015). Anger, in turn, is an important impelling factor of aggressive behavior (Leibenluft and Stoddard, 2013). The role of anger as an antecedent of aggression can be explained by the anger-related action tendency that is assumed to activate aggression-related motor impulses (Berkowitz and Harmon-Jones, 2004). Further, irritability can be conceptualized as a maladaptive response to frustration or threat (Leibenluft, 2011). Supporting this assumption, children in preschool age and in middle childhood who are prone to anger were found to be more likely to engage in aggressive behavior (e.g., Eisenberg et al., 1999; Arsenio et al., 2000; Olson et al., 2005; Wakschlag et al., 2015). The theoretical and empirical links between EF and anger on the one hand, and anger and aggression on the other hand suggest that the association of EF with aggression may partly be explained by the habitual experience of anger. So far, this assumption has received little attention, especially for the developmental period of middle childhood, and was therefore addressed in the present study.

With regard to different forms of aggression, most research has focused on relations of EF and physical rather than relational aggression. One reason for this may be that—as suggested by the frontal-lobe hypothesis of emotional and behavioral regulation outlined above—impairments in EF appear to be specific to physically aggressive behavior (Séguin, 2009). Those studies that included both forms of aggression revealed mixed findings. In line with the frontal-lobe hypothesis, EF was found to be negatively associated with physical aggression and not related with relational aggression in 3- to 6-year-old children (O'Toole et al., 2017). By contrast, other cross-sectional research has found negative relations of EF to both physical and relational aggression in early childhood; only working memory was positively associated with proactive relational aggression (Poland et al., 2016). However, these studies did not account for overlapping variance of physical and relational aggression, which may have an impact on the respective relations. With regard to middle childhood, previous research has failed to support the assumption that only physical aggression is related to deficits in EF. In a sample of fourth- and fifth-grade children, impaired central executive working memory, an indicator of EF, was associated with both physical and relational aggression (McQuade et al., 2013). Furthermore, in a population sample of 9-year-olds, working memory updating was negatively related only to relational, not to physical aggression (Granvald and Marciszko, 2016). Another study in middle childhood did not find significant paths of impaired EF to physical or relational aggression after controlling for symptoms of attention deficit/hyperactivity disorder (Diamantopoulou et al., 2007). Altogether, the inconsistency among studies that have taken both relational and physical aggression into account points to the need for further research into the role of EF in the development of different forms of aggression.

Regarding functions of aggression, few studies have differentiated between reactive and proactive aggression when examining the link between EF and aggression, particularly in middle childhood. In 9- to 12-year-old children, deficits in EF, particularly in response inhibition and planning, were found to be positively associated with reactive aggression. The relations between planning and reactive aggression, but not between planning and proactive aggression were moderated by hostile attributional biases (Ellis et al., 2009; Rathert et al., 2011). In addition, a measure of self-regulation that included EF-components was negatively linked to reactive, but not proactive aggression in 6- to 16-year-old children and adolescents (White et al., 2013). Thus, deficits in EF seem to be more involved in the development of reactive compared to proactive aggression. One explanation for the relation between deficits in EF and reactive aggression may be the potential mediating role of anger, as outlined above. Because anger is a major component of reactive, but not proactive aggression (for a review, see Hubbard et al., 2010), it can be assumed that only reactive aggression is indirectly predicted by poor EF via the experience of anger. Consequently, anger may also mediate between EF and reactive aggression.

### The current study

The aim of this study was to examine the prospective paths between EF and different forms (physical and relational) and functions (reactive and proactive) of aggression in a large population-based sample in middle childhood, with the habitual experience of anger considered as a potential underlying mechanism. The study included three measurement time-points covering 3 years. At T1, a latent factor of EF was calculated from measures of inhibition, set shifting, working-memory updating, and planning, which were assessed by using behavioral tasks and a teacher-report measure. Children's tendency to experience anger was assessed via parent reports at T1 and T2, and the forms and functions of aggression were rated by teachers at all three time points. The prospective paths were analyzed via structural-equation modeling, controlling for age, gender, and information-processing capacity.

Based on the theoretical assumptions and previous evidence outlined above, four hypotheses were postulated: First, we expected to find a negative relation between EF at T1 and physical aggression at T3, such that lower EF would predict higher levels of later physical aggression (Hypothesis 1). Considering relational aggression, the existing evidence is mixed, because some research found a negative (McQuade et al., 2013) and other either no (Diamantopoulou et al., 2007) or even a positive relation to EF (Poland et al., 2016). We therefore examined these competing predictions for the relation between EF at T1 and relational aggression at T3 in our model. In addition to potential direct effects, we expected negative indirect effects between EF at T1 and physical and relational aggression at T3 through habitual anger at T2 (Hypothesis 2). Thus, we proposed that lower EF would predict a higher tendency to experience anger at T2, which in turn would predict higher rates of physical and relational aggression at T3. With regard to the functions of aggression, we postulated that EF at T1 would be a negative predictor of reactive aggression at T3 but would be unrelated to proactive aggression at T3 (Hypothesis 3), based on earlier evidence (e.g., Ellis et al., 2009; Rathert et al., 2011). Furthermore, we expected negative indirect effects between EF at T1 and reactive aggression at T3 through habitual anger at T2. Thus, lower EF at T1 would predict a higher tendency to experience anger at T2, which in turn would predict higher rates of reactive aggression at T3 (Hypothesis 4).

In addition, we tested potential gender differences in the postulated paths between EF, anger, and aggression. In previous research, gender differences received little attention, in particular in conjunction with the distinction between forms and functions of aggression. Given the gender-related differences in the occurrence of the two forms of aggression (boys usually show more physical, girls slightly more relational aggression; e.g., Card et al., 2008), it was deemed important to include gender as a potential moderator in the analyses. However, the few studies that have addressed gender differences yielded little support for the assumption that the longitudinal links between EF and forms or functions of aggression might differ by gender (White et al., 2013). Based on these findings, we expected that the proposed associations would hold for boys and girls.

## Method

### Participants and procedure

The sample was part of a large longitudinal study on intrapersonal developmental risk factors in childhood and adolescence based at the University of Potsdam, Germany. The children were recruited from 33 public primary schools in the Federal State of Brandenburg, Germany. At T1, the sample consisted of *N* = 1,652 children (52.06% girls) aged between 6 and 11 years (*M* = 8.36, *SD* = 0.93)^1^. At T2, 1,611 children (51.8% girls) participated again (*M* = 9.12 years, *SD* = 0.93, range 7.11–11.89) and at T3, the remaining sample consisted of 1,501 children (51.5% girls; *M* = 11.07 years, *SD* = 0.92, range 9.12–13.76). This corresponds to a high retention rate of 97.5% from T1 to T2 and 92.3% from T2 to T3.

The mean interval between T1 and T2 was 9.14 months (*SD* = 1.80), and between T2 and T3, it was 23.83 months (*SD* = 1.66).

Approval for the procedure and the instruments was granted by the Ethics Committee of the authors' university as well as the Ministry of Education, Youth, and Sport of the Federal State of Brandenburg. The EF tasks were administered in individual test sessions by trained project members at the participants' schools. Parents and teachers completed the questionnaires either online or in paper-pencil form. For each child, informed consent was obtained from the parents.

### Measures

#### Executive function

The EF subcomponent *inhibition* was assessed by the Fruit Stroop task (Archibald and Kerns, 1999; adapted by Röthlisberger et al., 2010), a child-version of a Stroop paradigm with vegetables and fruits as stimulus items. The task consisted of four trials, and in each a page depicting 25 stimuli was presented to the child. Page 1 depicted colored rectangles (blue, green, red, yellow), page 2 showed fruits or vegetables in their typical colors (plum—blue, lettuce—green, strawberry—red, banana—yellow). Page 3 depicted the same fruits and vegetables, but all were colored gray. Page 4 displayed the same fruits and vegetables, but all were colored incorrectly. The child was instructed to name the correct color of the stimuli (pages 1 and 2), or to name the color that the fruits and vegetables should have (pages 3 and 4), as quickly as possible. For each page, the time (in seconds) required for giving correct responses for all 25 stimuli was measured. As dependent variable, an interference score was generated based on Röthlisberger et al. (2010): time p.4—[(time p.1 × time p.3)/(time p.1 + time p.3)]. Higher scores indicated a lower ability to successfully inhibit the prepotent response of naming the color in which the stimuli were depicted on page 4.

The EF subcomponent *working memory* was assessed using the Digit-Span Backward task (Petermann and Petermann, 2007). This is a complex working memory task (Best and Miller, 2010), and measures of complex working memory and updating have been found to be highly correlated in children (e.g., St Clair-Thompson and Gathercole, 2006). In this task, participants were told a sequence of digits, which they had to repeat in reverse order. Each trial consisted of two sequences of equal length. The first two sequences were 2 digits long, and in each of the next trials, the sequences were lengthened by one digit up to a maximum number of eight digits, yielding a total of 7 trials with 14 sequences. Within each trial, at least one sequence had to be answered correctly in order to proceed to the next trial. The dependent variable was the total number of sequences that had been repeated correctly with a potential range of 0 to 14.

The EF subcomponent *shifting* was assessed using the Cognitive Attention Shifting task (Röthlisberger et al., 2010; adapted from Zimmermann et al., 2002). Participants were presented with a single-colored fish and a multi-colored fish appearing simultaneously on the left- and right-hand side, respectively, of the computer screen. Children were told to feed each kind of fish and to always alternate between the two kinds by pressing one of two keys on a QWERTZ keyboard. Across several trials, the side on which the two kinds of fish appeared changed randomly. This required the children to remember their previous response—that is, which kind of fish they fed—in order to maintain the requirement of alternating feeding. A total of 46 trials (interstimulus intervals ranged from 300 to 700 ms) was separated by a short break during which positive feedback was given. The dependent variable was the number of correct responses for the 22 switch trials, that is, the trials that required children to change their response pattern (i.e., from alternately pressing left/right to repeating left/left or right/right; Austin et al., 2014).

The EF subcomponent *planning* was measured using items of the Planning and Organizing-scale from the Behavior Rating Inventory of Executive Function (BRIEF; Gioia et al., 2000). Eight of the original 10 items were selected based on their factor loadings and translated into German by two native speakers. The items covered a range of problems that students can face when they need to plan or organize present and future tasks for school (e.g., “does not plan tasks for school in advance”). Teachers indicated planning disability of their students during the past 6 months using a 5-point response scale ranging from 1 (*never)* to 5 (*always)*. A total score was computed by averaging the item scores. The internal consistency was high with α = 0.93.

#### Aggression

At all three time points, aggression was measured using a teacher-report questionnaire that contained subscales with three items each for physical and relational aggression as well as for proactive and reactive aggression.

The teachers first rated the frequency of physical and relational aggression during the past 6 months on a 5-point scale ranging from 1 (*never)* to 5 (*daily)* (physical aggression: e.g., “hit, shoved, or pushed peers”; relational aggression: e.g., “spread rumors or gossips about some peers”). The items were adapted from the Children's Social Behavior Scale—Teacher Form (CSBS-T; Crick, 1996). In a next step, teachers were asked to rate the *functions* of aggressive behaviors, based on the Instrument of Reactive and Proactive Aggression (IRPA; Polman et al., 2009; proactive aggression: e.g., “to be the boss,” reactive aggression: e.g., “because someone teased or upset him/her”). The response scale ranged from 1 (*never*) to 5 (*always*). The items on the function of aggression were only completed if the total score of the frequency of physical and relational aggression was larger than 1. Thus, the children for whom the teachers reported no physical or relational aggression at all had logical missing values on the measures of proactive and reactive aggression. The handling of these missing values is explained below.

For all four subscales, total scores were created by averaging the corresponding items, based on acceptable to high internal consistencies (physical aggression: α_t1_ = 0.93, α_t2_ = 0.94, α_t3_ = 0.93; relational aggression: α_t1_ = 0.91, α_t2_ = 0.92, α_t3_ = 0.91; proactive aggression: α_t1_ = 0.80, α_t2_ = 0.77, α_t3_ = 0.81; reactive aggression: α_t1_ = 0.85, α_t2_ = 0.84, α_t3_ = 0.88).

#### Habitual anger

At T1 and T2, parents rated the extent to which their children habitually experienced anger with the subscale Anger/Frustration of the Temperament in Middle Childhood Questionnaire (TMCQ; Simonds, 2006). This subscale assesses the extent of anger shown by the child in response to the interruption of ongoing tasks or goal blocking (e.g., “my child gets angry when she or he has trouble with a task”). The scale contains 7 items, and the response scale ranges from 1 (*almost always untrue*) to 5 (*almost always true*). A total score was obtained by averaging the item scores. The internal consistency was good with α = 0.80 at both time points.

#### Information-processing capacity

Information-processing capacity was assessed at T1with the Digit-Symbol Test (DST) of the German version of the Wechsler Intelligence Scale for Children (Petermann and Petermann, 2007). Children were given a worksheet on which they had to assign either common shapes (Version A; ages 6–7) or the numbers 1 to 7 (Version B; ages 8 and older) to various symbols. A key in which a specific shape/number was paired with each of the symbols was presented in the first row of the worksheet. For both versions, the number of correct symbols allocated within 120 s was measured (standardized *T*-values were calculated). Information-processing capacity was measured to control for basic intellectual ability, which could be confounded with EF.

### Plan of analysis

SPSS (Version 23) was used for descriptive computations, and the hypotheses were analyzed through structural equation models using Mplus (Version 7.4; Muthén and Muthén, 2012). In all models, the robust Maximum Likelihood estimator (MLR) was used to account for the non-normal distribution of the data. Missing data were handled by the Full Information Maximum Likelihood (FIML) estimation option to avoid a reduction in sample size. To be able to use the FIML approach for the logical missings on the items of the functions of aggression, we included a participant's overall frequency scores of aggression at all three time points as covariates in the models. The frequency of aggression is a perfect predictor of the presence or absence of a data point on the two functions of aggression. Therefore, missing data could be treated as missing at random, which allowed us to use the FIML approach (Enders, 2010).

Because participants were nested within school classes, class was included as a cluster variable in all analyses. Due to the trait-like nature of aggressive behavior, we included a random intercept for both forms and both functions of aggression, following the recommendation of Hamaker et al. (2015). Because habitual anger and the three EF subcomponents were not assessed at all three time points, we were not able to include random intercepts for these variables (at least three time points are required to specify random intercepts). EF was modeled as a latent factor using the measures of *working memory, inhibition, shifting*, and *planning* as indicators.

The model fits of the measurement model of EF and of the structural equation models were evaluated based on the criteria of Hu and Bentler (1998), with a comparative fit index (CFI) ≥0.95, a root-mean-square error of approximation (RMSEA) ≤ 0.06, and a standardized root-mean-square residual (SRMR) ≤ 0.08 indicating a good fit. The χ^2^-statistic was not interpreted as a measure of absolute fit, because it is biased in large samples (Schermelleh-Engel et al., 2003). Bootstrap analyses were used to test indirect effects. If the bootstrapped 95% confidence interval does not include zero, the indirect effect is considered to be significant (Shrout and Bolger, 2002). The potential moderating effect of gender was examined using multi-group analyses. The measurement invariance between the gender groups was assessed based on comparisons between a fully constrained and a fully unconstrained (freed) model. The indicator for measurement invariance was a nonsignificant difference in χ^2^ with scale corrections for the MLR estimator, as proposed by Satorra and Bentler (2001) or a nonsignificant Wald test for invariance in the indirect effects.

## Results

### Descriptive statistics, gender differences, factor analysis, and correlations

The descriptive statistics of all study variables are presented in Table 1. Gender differences were analyzed using *t*-tests for independent samples. If the assumption of homogeneity of variance was violated (as indicated by the Levene's Test for Equality of Variances), the degrees of freedom were adjusted using the Welch-Satterthwaite method. To account for multiple testing, we used a strict alpha level of *p* < 0.01. Effect size was calculated as Cohen's *d*. Boys were rated to be significantly more physically aggressive than were girls at all time points, all *t*s ≥ 11.00, *p*s < 0.001, *d*s ≥ 0.60. For relational aggression, no significant gender differences were found. Boys as compared to girls were also rated to show significantly more proactive aggression at T1 and T3, all *t*s ≥ 3.48, *p*s < 0.01, *d*s ≥ 0.25, and more reactive aggression at T2 and T3, all *t*s ≥ 3.48, *p*s < 0.01, *d*s ≥ 0.30. For the measure of anger, a significant gender difference was found only at T1, with higher scores for boys than for girls, *t*_(1332)_ = 3.64, *p* < 0.001, *d* = 0.19.

**Table 1:** Descriptive statistics of the study variables for the total sample and for boys and girls.

	***n***	**Range**	**Total**	**Boys**	**Girls**
			***M* (*SD*)**	***M* (*SD*)**	***M* (*SD*)**
**EF T1**					
Updating	1,635	0–16	6.18 (1.46)	6.21 (1.47)	6.16 (1.45)
Shifting	1,555	0–22	18.16 (3.92)	**17.77 (3.94)**	**18.52 (3.86)**
Inhibition	1,640	7–89^a^	24.91 8.76)	**25.65 (9.48)**	**24.24 (7.99)**
Planning	1,417	1–5	2.30 (0.89)	**2.50 (0.90)**	**2.11 (0.85)**
**ANGER**					
T1	1,334	1–5	2.63 (0.74)	**2.71 (0.74)**	**2.56 (0.72)**
T2	1,190	1–5	2.75 (0.71)	2.62 (0.72)	2.53 (0.70)
**FORMS OF AGGRESSION**					
T1 Physical A	1,408	1–5	1.49 (0.79)	**1.79 (0.91)**	**1.21 (0.52)**
T2 Physical A	1,145	1–5	1.48 (0.77)	**1.79 (0.90)**	**1.18 (0.46)**
T3 Physical A	1,104	1–5	1.43 (0.75)	**1.67 (0.88)**	**1.20 (0.49)**
T1 Relational A	1,405	1–5	1.50 (0.70)	1.54 (0.70)	1.47 (0.70)
T2 Relational A	1,144	1–5	1.56 (0.76)	1.61 (0.79)	1.51 (0.73)
T3 Relational A	1,102	1–5	1.50 (0.71)	1.55 (0.76)	1.45 (0.66)
**FUNCTIONS OF AGGRESSION**					
T1 Reactive A	756	1–5	2.11 (0.92)	2.81 (0.95)	2.63 (0.97)
T2 Reactive A	627	1–5	2.17 (0.89)	**3.00 (0.97)**	**2.63 (0.93)**
T3 Reactive A	568	1–5	2.00 (0.95)	**2.56 (1.09)**	**2.25 (1.00)**
T1 Proactive A	753	1–5	2.73 (0.96)	**2.21 (0.96)**	**1.98 (0.83)**
T2 Proactive A	621	1–5	2.85 0.98)	2.22 (0.90)	2.08 (0.86)
T3 Proactive A	568	1–5	2.42 (1.06)	**2.18 (0.99)**	**1.77 (0.81)**
DST T1	1,644	27–80^a^	51.28 (9.17)	**49.61 (8.97)**	**52.81 (9.08)**

EF, executive function; A, aggression; DST, digit-symbol test;

a *Max values are theoretically infinite, thus, table values are sample-specific. Means in bold differ significantly between boys and girls*.

With regard to EF, girls scored significantly higher than did boys on shifting, *t*_(1535)_ = 3.79, *p* < 0.001, *d* = 0.19, and boys scored significantly higher than did girls on inhibition, *t*_(1534.04)_ = 3.24, *p* < 0.01, *d* = 0.16, and planning, *t*_(1415)_ = 8.23, *p* < 0.001, *d* = 0.56. Furthermore, girls scored significantly higher than did boys on information-processing capacity, *t*_(1642)_ = 7.18, *p* < 0.001, *d* = 0.35. Due to these differences, gender was included as a covariate in the structural equation models (except for the multi-group model).

A latent factor of EF was specified by using the measures of working memory, inhibition, shifting, and planning as manifest indicators. Factor loadings were of moderate size (working memory: 0.55, inhibition: −0.62, shifting: 0.46, planning: −0.51; all *p*s <.001). The resulting measurement model showed a good fit (χ^2^[3] = 2.76, *p* = 0.25; CFI = 1.00; RMSEA = 0.02, 90% CI [0.00,0.05]; SRMR = 0.01).

Table 2 presents the correlations among all study variables and their links with age and information-processing capacity. The following significant correlations were found: EF at T1 was negatively correlated with anger at T1 and T2, and with all aggression measures at T1, T2, and T3. Anger was positively correlated with all aggression measures within and across time points. Age and information-processing capacity were positively correlated with EF. Furthermore, age was negatively correlated with reactive aggression at T3, and information-processing capacity was negatively correlated with physical aggression at all time points and with reactive aggression at T1 and T3. As a consequence, we decided to include age and information-processing capacity as covariates in all models.

**Table 2:** Correlations between executive function, anger, aggression, and age.

	**1**	**2**	**3**	**4**	**5**	**6**	**7**	**8**	**9**	**10**	**11**	**12**	**13**	**14**	**15**	**16**	**17**
T1 EF		−0.19^***^	−0.23^***^	−0.34^***^	−0.33^***^	−0.28^***^	−0.24^***^	−0.19^***^	−0.24^***^	−0.25^***^	−0.27^***^	−0.33^***^	−0.15^**^	−0.18^**^	−0.16^**^	0.42^***^	0.45^***^
T1 Anger			0.70^***^	0.19^***^	0.22^***^	.12^***^	0.16^***^	0.15^***^	0.09^***^	0.11^**^	0.18^**^	0.11^**^	0.09^*^	0.08	0.08^*^	−0.04	−0.01
T2 Anger				0.17^***^	0.20^***^	.16^***^	0.18^***^	0.13^***^	0.13^***^	0.12^**^	0.12^***^	0.11^*^	0.11^**^	0.08^*^	0.08^*^	−0.02	−0.06
T1 Phy A					0.71^***^	0.48^***^	0.62^***^	0.45^***^	0.35^***^	0.28^***^	0.23^***^	0.21^***^	0.44^***^	0.30^***^	0.26^***^	−0.13^***^	−0.02
T2 Phy A						0.50^***^	0.44^***^	0.65^***^	0.37^***^	0.19^***^	0.28^***^	0.26^***^	0.34^***^	0.40^***^	0.28^***^	−0.10^***^	−0.03
T3 Phy A							0.33^***^	0.30^***^	0.67^***^	0.14^***^	0.16^***^	0.13^***^	0.24^***^	0.23^***^	0.32^***^	−0.12^***^	−0.01
T1 Rel A								0.53^***^	0.37^***^	0.19^***^	0.13^***^	0.13^***^	0.40^***^	0.25^***^	0.19^***^	−0.05	−0.05
T2 Rel A									0.30^***^	0.11^***^	0.21^***^	0.17^***^	0.30^***^	0.39^***^	0.21^***^	0.02	−0.04
T3 Rel A										0.07^**^	0.11^***^	0.18^***^	0.23^***^	0.23^***^	0.36^***^	−0.04	−0.02
T1 Reac A											0.43^***^	0.21^***^	0.26^***^	0.12^***^	0.10^*^	−0.06^*^	−0.05
T2 Reac A												0.36^***^	0.18^***^	0.19^***^	0.15^**^	−0.07	0.04
T3 Reac A													0.17^***^	0.19^***^	0.35^***^	−0.11^**^	−0.16^**^
T1 Proac A														0.44^*^	0.33^*^	0.01	0.02
T2 Proac A															0.35^*^	−0.01	0.04
T3 Proac A																0.02	−0.00
DST																	0.00
T1 Age																	

*EF, executive function; Phy A, physical aggression; Rel A, relational aggression; Reac A, reactive aggression; Proac A, proactive aggression; DST, digit-symbol test*.

*** *p < 0.001*,

** *p < 0.01*,

* *p < 0.05*.

### Hypothesis-testing analyses

**Figure 1:**
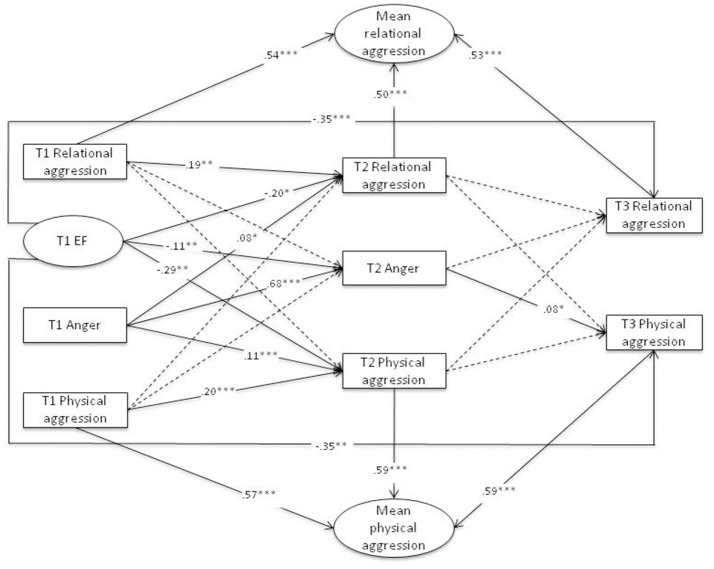
Prediction of the Forms of Aggression. Standardized path coefficients are displayed; dotted lines = nonsignificant path coefficients; mean physical and mean relational aggression partial out between-person stability over time (random intercept); model fit: χ^2^(39) = 363.05, *p* < 0.001; CFI = 0.93; RMSEA = 0.07, 90% CI = [0.06, 0.08]; SRMR = 0.05; for clarity of presentation, only paths between the time points are presented in the figure, but within-time correlations at all time points were also included in the model. ^***^p < 0.001, ^**^p < 0.01, ^*^p < 0.05.

**Figure 2:**
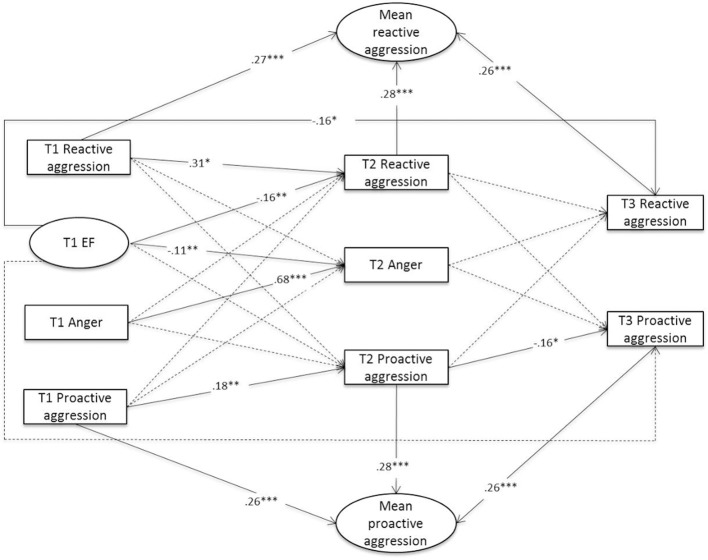
Prediction of the Functions of Aggression Notes*:* Standardized path coefficients are displayed; dotted lines = nonsignificant path coefficients; mean proactive and mean reactive aggression partial out between-person stability over time (random intercept); model fit: χ^2^(61) = 407.04, *p* < 0.001; CFI = 0.91; RMSEA = 0.06, 90% CI = [0.05, 0.06]; SRMR = 0.05; for clarity of presentation, only paths between the time points are presented in the figure, but within-time correlations at all time points were also included in the model. ^***^p < 0.001, ^**^p < 0.01, ^*^p < 0.05.

Two separate models were specified to examine the proposed links between EF, anger, and aggression: one for the forms of aggression (physical and relational; see Figure 1) and one for the functions of aggression (reactive and proactive; see Figure 2). Age, gender, and information-processing capacity were included as covariates in both models.

#### Links between EF, anger, and forms of aggression

The model for the forms of aggression (Figure 1) showed an acceptable model fit (χ^2^[39] = 363.05, *p* < 0.001; CFI = 0.93; RMSEA = 0.007, 90% CI [0.06,0.08]; SRMR = 0.05). In line with Hypothesis 1, we found that controlling for stable individual differences in aggression, there was a significant negative path from EF at T1 to physical aggression at T3. Regarding relational aggression at T3, our data revealed a significant negative link to EF at T1 as well. Thus, the lower a child's EF, the higher the teacher-rated frequency of physical and relational aggression after the 3-year period. The paths from EF at T1 to physical and relational aggression at T2 were also negative and significant.

A significant negative link between EF at T1 and habitual anger at T2 was found, indicating that the lower children's EF was at T1, the more anger-prone they were rated by their parents at T2. Moreover, there was a significant positive link between habitual anger at T1 and both physical and relational aggression at T2, and there was a significant positive path from habitual anger at T2 to physical aggression at T3, but no significant link to relational aggression at T3.

Hypothesis 2 postulated indirect negative effects between EF at T1 and both forms of aggression at T3 through habitual anger at T2. This hypothesis was only partially confirmed, because an indirect effect was found only for physical aggression, β = −0.01, 95% CI [−0.021, −0.001]. For relational aggression, the indirect path was not found due to the nonsignificant path from habitual anger at T2 to relational aggression at T3 Links between EF, Anger, and Functions of Aggression.

The model for the functions of aggression (Figure 2) also showed an acceptable fit (χ^2^[61] = 407.04, *p* < 0.001; CFI = 0.91; RMSEA = 0.06, 90% CI [0.05,0.06]; SRMR = 0.05). As predicted in Hypothesis 3, we found that controlling for stable individual differences in aggression, there was a significant negative path from EF at T1 to reactive aggression at T3, whereas the path from EF at T1 to proactive aggression at T3 was nonsignificant. Similarly, there was a significant negative link between EF at T1 and reactive, but not proactive aggression at T2. Finally, Hypothesis 4 postulated an indirect negative effect between EF at T1 and reactive aggression at T3 through habitual anger at T2. This hypothesis was not supported, because no significant link between T2 habitual anger and T3 reactive aggression was found.

### Multi-group analyses of potential gender differences

To examine potential gender differences in our first model, considering physical and relational aggression, we compared a fully unconstrained model, in which all paths were allowed to vary between girls and boys [fit: χ^2^_(72)_ = 396.54, *p* < 0.001; CFI = 0.92; RMSEA = 0.07, 90% CI [0.07,0.08]; SRMR = 0.05], with a fully constrained model, in which all paths were constrained to be equal [fit: χ^2^_(132)_ = 650.12, *p* < 0.001; CFI = 0.88; RMSEA = 0.07, 90% CI [0.06,0.07]; SRMR = 0.12]. The difference in χ^2^ was significant, Δχ^2^_(60)_ = 262.66, *p* < 0.001, indicating gender differences in specific parts of the model. Therefore, we computed a revised model [fit: χ^2^_(121)_ = 442.26, *p* < 0.001; CFI = 0.92; RMSEA = 0.06, 90% CI [0.05,0.06]; SRMR = 0.07], in which we constrained all paths of the model to be equal between boys and girls, but with free estimation of those covariances and intercepts that were found to be different between boys and girls (e.g., the covariances between physical and relational aggression at all three time-points). The revised model had a significantly better fit than the fully constrained model, Δχ^2^_(11)_ = 150.69, *p* < 0.001, and did not fit significantly worse than the fully unconstrained model, Δχ^2^_(49)_ = 66.0, *p* = 0.053. In the revised model, there were no significant gender differences in the hypothesized paths [all Δχ^2^s ≤ 3.64*, ps* ≥ 0.19; all *W*(1) ≤ 0.13, *ps* ≥ 0.72].

For our second model, considering reactive and proactive aggression, we followed the same procedure. We also compared a fully unconstrained model, in which all paths were allowed to vary between girls and boys [fit: χ^2^_(116)_ = 445.94, *p* < 0.001; CFI = 0.91; RMSEA = 0.06, 90% CI [0.05,0.06]; SRMR = 0.05], with a fully constrained model [fit: χ^2^_(198)_ = 624.94, *p* < 0.001; CFI = 0.088; RMSEA = 0.05, 90% CI [0.05,0.06]; SRMR = 0.08]. The difference in χ^2^ was significant, Δχ^2^_(82)_ = 188.41, *p* < 0.001. Then, we computed a revised model [fit: χ^2^_(191)_ = 524.07, *p* < 0.001; CFI = 0.91; RMSEA = 0.05, 90% CI [0.04,0.05]; SRMR = 0.06], in which we constrained all paths of the model to be equal between boys and girls, but with free estimation of some covariances and intercepts that were found to be different (for instance the covariances between the overall frequency scores of aggression at the three time-points). The revised model had a significantly better fit than the fully constrained model, Δχ^2^_(7)_ = 86.93, *p* < 0.001, and did not fit significantly worse than the fully unconstrained model, Δχ^2^_(75)_ = 91.87, *p* = 0.090. In the revised model, there were no significant gender differences in the hypothesized paths [all Δχ^2^s ≤ 2.79; *ps* ≥.095; all *W*(1) ≤ 0.63, *ps* ≥ 0.43].

## Discussion

The aim of the present study was to examine the longitudinal associations of EF (calculated as a latent factor of EF from behavioral measures of inhibition, set shifting, and working-memory updating, as well as teacher-reported planning), parent-reported habitual anger, and teacher-reported forms of aggression (i.e., physical and relational) and functions of aggression (i.e., proactive and reactive) in middle childhood. The hypotheses were examined in a large population-based sample in a three-wave design over a period of 3 years.

In line with Hypothesis 1, we found that EF was a significant negative predictor of physical aggression, and we also found a significant negative path between EF and relational aggression. This was true for the paths from T1 to T2 as well as from T1 to T3. Thus, the more deficits in EF children showed at T1 the higher was their teacher-rated frequency of both forms of aggression 1 and 2 years later. Our results held after controlling for information-processing capacity, gender, and age in the whole model. Further, we controlled for stable between-person differences by inclusion of a random intercept for forms and functions of aggression. Following the reasoning by Hamaker et al. (2015), this method allowed us to uncover causal relationships in within-persons processes. Therefore, our findings replicate longitudinal findings from other age groups (e.g., Hughes et al., 1998; Hughes and Ensor, 2008; Ogilvie et al., 2011; Schoemaker et al., 2013; Olson et al., 2017), and they extend previous cross-sectional research in middle childhood (e.g., McQuade et al., 2013), showing that lower EF at a mean age of 8 years predicted higher physical and relational aggression at about 9 and 11 years as within-person change. Furthermore, the negative path from EF to physical aggression is consistent with the frontal-lobe hypothesis of physical aggression (Séguin, 2009), and the social information-processing theory of aggression (Crick and Dodge, 1994). Consequently, the children in our study, who showed physically and relationally aggressive behavior at the age of 9 and 11 years might already have manifested significant cognitive deficits in their EF abilities, located in the frontal lobe, at the age of 8 years.

Our finding of significant negative paths from EF at T1 to relational aggression at both T2 and T3 confirms some of the previous cross-sectional findings (McQuade et al., 2013), but contradicts others (no significant path: Diamantopoulou et al., 2007; positive relation: Poland et al., 2016). Nevertheless, our finding is in line with the social information-processing theory of aggression that proposes that physically as well as relationally aggressive children have deficits in their cognitive processing of social situations (Crick and Dodge, 1994). To further examine the role of EF in the development of relational aggression, it may be important to include a more differentiated assessment of relational aggression that considers the complexity of different relationally aggressive behaviors. According to Crick et al. (1999), relationally aggressive behaviors can take different forms ranging from relatively simple, direct types (e.g., threatening to end the friendship) to more complex, indirect types (e.g., mobilizing peer group members against a certain child to make that child feel excluded). The latter requires a higher level of cognitive skills to be used effectively. Thus, it may be that only the direct types of relational aggression are related to deficits in EF, whereas the indirect types of relational aggression are unrelated or even positively related to EF. However, this remains to be tested in future studies.

In Hypothesis 2, we postulated that anger would mediate the link between EF and both forms of aggression. However, this prediction was only confirmed for physical, and not for relational aggression. For physical aggression, the results indicate that the lower the children's EF at T1 the higher their parent-reported habitual anger was at T2, which in turn predicted more teacher-rated physical aggression at T3. Even though the size of the indirect effect was small, it was still significant after controlling for age, gender, information-processing capacity, and stable between-person differences of physical and relational aggression. The tendency to experience anger is only one of many intrapersonal factors involved in the complex emergence of aggressive behavior (see Krahé, 2013, for a review). However, our findings extend previous cross-sectional research (e.g., de Castro et al., 2005) and support the role of anger as one explanatory construct in the link between EF and physical aggression in middle childhood. Further, the negative mediation between EF and physical aggression by anger highlights the importance of considering emotions in the social-cognitive information processing of children who display aggressive behavior (Lemerise and Arsenio, 2000). Our study is—as far as we know—the first to uncover this meditational effect over a time-span of 3 years in middle childhood, which underlines the need to consider large time intervals in the development of physical aggression, as well as both emotional and cognitive processes.

For relational aggression, this mediation effect was not found. This finding is inconsistent with theory and previous research that has found anger to be involved in the development of both physical and relational aggression (Crick et al., 1999, 2002). However, the positive path from anger at T2 to relational aggression at T3 only narrowly missed the level of significance (*p* = 0.054, β = 0.06). This trend tentatively suggests that the tendency to experience anger may be involved in the negative link between EF and later relational aggression. To date, only few studies have differentiated between forms of aggression when examining the link between anger and aggression. Future research is needed to explore potential differences between relational and physical aggression regarding the association with EF and anger, not only in middle childhood.

With regard to the functions of aggression, we found that EF was a significant negative predictor of reactive aggression, but that EF was unrelated to proactive aggression. This pattern is consistent with Hypothesis 3 and was found for the paths from EF at T1 to functions of aggression both at T2 and at T3. It is in line with the few previous cross-sectional studies regarding the association between EF and the functions of aggression in middle childhood (e.g., Ellis et al., 2009; White et al., 2013). Moreover, the nonsignificant path from EF to proactive aggression is consistent with research on CU traits, which are conceptually linked to proactive aggression (e.g., Pardini et al., 2003). Several studies have shown that CU traits are unrelated to deficits in different domains of EF such as inhibition (Tye et al., 2017) or set shifting (Mitchell et al., 2002). Furthermore, our findings support the theoretical differentiation of the two functions of aggression. Higher EF enables children to behave in a planned and deliberate fashion, which is characteristic of proactive aggression. In contrast, reactive aggression refers to impulsive aggressive acts that do not require sophisticated planning. Thus, the inability to plan and to inhibit behavioral responses, both components of low EF, may explain the direct negative paths between EF and reactive aggression at 1 or 2 years later found in this study.

Contrary to Hypothesis 4, no indirect link between EF and reactive aggression via anger was found. We did find a negative link between EF and anger, confirming previous evidence that EF is involved in the regulation of negative affect in children (e.g., Carlson and Wang, 2007). This finding is also consistent with research on deficits in processes associated with EF, for instance error monitoring, in children with chronic irritability (Perlman et al., 2015). However, there was no link between anger and reactive aggression, which is surprising given that anger is assumed to be a major impelling factor of reactive aggression during childhood (e.g., Eisenberg et al., 1999; Arsenio et al., 2000; Olson et al., 2005; Leibenluft and Stoddard, 2013). One explanation may be that the relation between anger and reactive aggression depends on moderating variables. These may include the ability to regulate the behavioral impulses related to anger, or the tendency to act impulsively, which relates to less voluntary and more reactive aspects of control (Rothbart and Rueda, 2005). Another reason could be that until now the relations of chronic anger, irritability, and disruptive behavior symptoms were mainly investigated in clinical samples with no differentiation between reactive and proactive aggression (e.g., Wakschlag et al., 2015). In addition, further variables besides anger may act as mediators of the link between EF and reactive aggression. These may include social information-processing variables, such as theory of mind (Renouf et al., 2010) or hostile attribution bias (de Castro et al., 2002).

In addition to the examination of the longitudinal links between EF and aggression, our study contributes to previous research by considering potential gender differences. We did find gender differences on some of the study variables, in particular on the frequency of physical aggression, with boys scoring higher than girls at all three time points. This finding is in line with a meta-analysis on gender differences in aggressive behavior (Card et al., 2008). However, the multi-group analyses revealed that the predictive paths from EF to aggression and from EF to habitual anger as well as the indirect paths from EF over anger to aggression did not vary by gender, which confirms and extends previous cross-sectional research (White et al., 2013). Consequently, the processes and mechanisms that lead from EF to aggressive behavior seem to be equivalent in girls and boys in middle childhood.

### Strengths and limitations

We believe that the present study has several strengths. These include the large sample size, the longitudinal design with three time points covering about 3 years across middle childhood, the differentiation of forms and functions of aggression, and the examination of potential gender differences. Furthermore, we used three different sources to assess the study constructs, a procedure that is known to reduce common method biases (Podsakoff et al., 2003). A final strength is the inclusion of a random intercept, by which we controlled for the stability of individual differences in the forms and functions of aggressive behavior. This new procedure is recommended to overcome the limitations of the traditional cross-lagged model by separating within-person change, which is the focus of our models, from stable between-person differences (Hamaker et al., 2015).

Despite these strengths, some limitations have to be noted. One refers to the distinction between “cool” and “hot” aspects of EF during childhood and adolescence (Zelazo and Carlson, 2012). Cool EF is usually associated with the lateral prefrontal cortex and operates in relation to more abstract and decontextualized problems. In contrast, hot EF is associated with the orbitofrontal cortex and operates in more motivationally and emotionally significant situations (Zelazo and Müller, 2002). The measures we used to assess EF covered only the cool component. Nevertheless, our findings demonstrate that cool EF alone seems to play an important role in the prediction of physical, relational, and reactive aggression in middle childhood. A further limitation of our study is that aggression was only assessed by teacher reports. This may have led to an underestimation of relationally aggressive behavior because it includes more indirect forms of aggression that may be less obvious for teachers. Thus, peer reports or peer nominations could provide important additional information because aggressive behavior usually takes place within the peer group. Finally, it is important to mention that our sample was a community sample. Whether similar associations are also found for clinical populations of youth with serious levels of aggression and/or chronic symptoms of irritability is a question for future research.

## Conclusion and implications

Our study extends the existing literature about the relation between EF and aggression in middle childhood by taking the habitual tendency to experience anger as a potential mediator into account, by considering different forms and functions of aggressive behavior, and by analyzing gender differences. We found that EF predicted physical, relational, and reactive aggression over the course of middle childhood, and that the link between EF and physical aggression was partly mediated by habitual proneness to anger. Although there were gender differences in the frequency of aggressive behavior, these predictive paths were not moderated by gender. Our findings highlight the importance of addressing EF in programs that aim to reduce aggressive behavior. In the last years, an increasing number of programs to promote EF has been developed, and the effectiveness of these programs has been demonstrated (Diamond and Lee, 2011), making EF a promising candidate for the prevention of aggression. For the prevention of physical aggression in particular, teaching strategies for coping with anger should also be considered. Regarding gender, our findings indicate that both gender groups should be addressed in prevention programs. Although the mean level of physically aggressive behavior in particular may be higher among boys, the paths between EF, anger, and aggression seem to be similar for girls and boys. Our study underlines the need to promote the development of EF not only in early, but also in middle childhood to prevent later physical, relational, and reactive aggression and its negative consequences.

## Author contributions

All authors have contributed substantially to the conceptualization and the design of the work. HR, AH, and FK: Have primarily collected, analyzed and interpreted the data; HR and AH: Have written the first draft of the paper, and all authors contributed to revise the paper; BK and BE: Have provided input and supervision to the analyses. All authors agreed to be accountable for all aspects of the work.

### Conflict of interest statement

The authors declare that the research was conducted in the absence of any commercial or financial relationships that could be construed as a potential conflict of interest.

## Footnotes

^1^The original sample size was *N* = 1,657, however in the present study, 5 children were excluded due to missing values on the cluster variable that was included in the analyses (see section 2.3. Plan of Analyses).
